# A Legume Host Benefits More from Arbuscular Mycorrhizal Fungi Than a Grass Host in the Presence of a Root Hemiparasitic Plant

**DOI:** 10.3390/microorganisms10020440

**Published:** 2022-02-15

**Authors:** Xiaolin Sui, Kaiyun Guan, Yan Chen, Ruijuan Xue, Airong Li

**Affiliations:** 1 Yunnan Key Laboratory for Wild Plant Resources, Department of Economic Plants and Biotechnology, Chinese Academy of Sciences, Kunming 650201, China; suixiaolin@mail.kib.ac.cn (X.S.); guanky@mail.kib.ac.cn (K.G.); chenyan@mail.kib.ac.cn (Y.C.); xueruijuan@mail.kib.ac.cn (R.X.); 2 Yunnan Key Laboratory for Fungal Diversity and Green Development, Kunming Institute of Botany, Chinese Academy of Sciences, Kunming 650201, China; 3 Key Laboratory of Biogeography and Bioresource in Arid Land, Xinjiang Institute of Ecology and Geography, Chinese Academy of Sciences, Urumqi 830011, China

**Keywords:** host-parasite interaction, photosynthesis, AMF, mineral nutrient, biomass

## Abstract

In nature, most plants parasitized by root hemiparasites are also colonized by mutualistic arbuscular mycorrhizal (AM) fungi, highlighting the prevalence of this tripartite interaction. AM colonization is generally found to improve the growth of parasitized legumes but has little impact on grass hosts parasitized by root hemiparasites, and the underlying mechanisms are still unclear. In this study, we conducted a pot experiment to test the influence of AM fungus (*Glomus mosseae*) on the growth and photosynthesis of leguminous *Trifolium repens* and gramineous *Elymus nutans* in the presence of a root hemiparasitic plant (*Pedicularis kansuensis*). The results showed that inoculation with AM fungi significantly improved the growth performance of parasitized legumes via enhancing their nutrient status and photosynthetic capacity, even though a larger *P. kansuensis* parasitized the legume host in the AM treatment. In contrast, AM colonization slightly improved the shoot DW of grass hosts by suppressing haustoria formation and the growth of *P. kansuensis*. Our results demonstrated that legume hosts benefit more from AM inoculation than grass hosts in the presence of hemiparasitic plants, and set out the various mechanisms. This study provides new clues for parsing the tritrophic interaction of AM fungi, parasitic plants, and host plants.

## 1. Introduction

Root hemiparasitic plants are globally important, as they are widely distributed in almost all ecosystems and have profound effects on community productivity, community structures, community diversity, and nutrient cycling [1,2,3,4]. Despite their normal appearance with green leaves and branching roots, root hemiparasitic plants depend largely on host plants to acquire nutrients via parasitic organs called haustoria [5,6]. Hemiparasitic plants typically deprive mineral nutrients, water, and carbohydrates from their host plants and can cause significant biomass loss or reproductive reduction in host plants [7,8,9]. In view of the intimate relationship between hosts and hemiparasites, studies of parasitic plants have focused on host-parasite interactions and the influencing factors. To our knowledge, a majority of studies have investigated the effect of abiotic factors (e.g., nutrient supply, water availability, etc.) [7,10,11,12,13] on regulating the impact of hemiparasite on host performance, but only a few studies have addressed the roles played by microorganisms (e.g., arbuscular mycorrhizal fungi) [14,15].

Arbuscular mycorrhizal (AM) fungi are common soil microflora components and can form a mutualistic relationship with more than 80% of plant species in terrestrial ecosystems [16]. Hemiparasitic plants are also widely distributed and have a wide range of host species [3], which generally occur with AM fungi coincidence. For instance, most plants that can be parasitized by hemiparasites are also colonized by AM fungi, highlighting the prevalence of this tripartite interaction in nature. As nutrients are the primary demands of root hemiparasitic plants for host plants, nitrogen and phosphorous supply have been proven to regulate the interaction between host and parasite [7,12,13]. Through their positive effects on enhancing plant nutrient status (e.g., N and P) [17,18], similar to nutrient supply, AM fungi play a crucial role in altering the degree of parasite performance and its impact on a particular host [15]. For instance, Davies and Graves [7] found that inoculation with *Glomus* spp. significantly improved the growth of *Rhinanthus minor*, but had no impact on its grass host *Lolium perenne*. Salonen et al. [14] found that inoculation with *G. lamellosum* or *G. mosseae* affected neither the growth of hemiparasite *Odonites vulgaris* nor the grass host *Poa annua*. In contrast, the growth of the legume host *Trifolium repens* and hemiparasite *R. serotinus* were all improved by *G. clarodium*. Similarly, Sui et al. [15] found that inoculation with *G. mosseae* greatly enhanced the growth performance of both hosts and parasites in legume *T. repens*-*Pedicularis rex/tricolor* pairs. Based on the limited number of studies, we found that although AM colonization had inconsistent effects on the growth of attached parasites, it stably and negligibly affected the grass hosts. In contrast, AM colonization significantly improved the growth performance of legume hosts, even though a larger hemiparasite was attached to them. Although no studies have clarified the different effects of AM fungi on the growth performance of parasitized host plants, we should also keep in mind that host identity and its responses to AM fungi may be significant reasons.

Grass and legume plants are preferred hosts for hemiparasitic plants but have different responses to AM fungi. Grasses often form a more branched and more exploitative root system, and therefore are likely to benefit less from AM fungi [19]. In contrast, legumes are often highly dependent on AM fungi [20,21]. Hence, we hypothesized that higher AM responsive hosts might benefit more from AM colonization than lower AM responsive hosts. However, previous studies used different parasites, AM species, and soil conditions, which are all essential factors that can impact the effect of AM fungi on host growth [22,23,24]. This makes it difficult to clarify whether host identity and its response to AM colonization determine the influence of AM fungi on the growth of parasitized host plants. Stein et al. [25] compared the impact of AM species on the growth of various host species parasitized by *R. minor* under the same soil conditions. The results confirmed that biomasses of the parasitized legumes *Vicia cracca* and *T. pretense* were more significantly improved by AM fungi than the parasitized grass *Festuca rubra*. However, in this study, parasitism induced by *R. minor* did not affect the growth of these two legume hosts, but significantly affected the growth of the grass host in nonmycorrhizal pots. Therefore, it is unclear whether the inconsistent impacts of AM colonization on parasitized legume or grass hosts could be explained by the different responses of host plants to AM colonization or to parasitism.

In this study, we compared the effect of AM fungi on the performance of two different AM responsive hosts parasitized by the root hemiparasite *Pedicularis kansuensis* via a manipulated pot experiment with the same growth conditions. Here, the low AM responsive grass *Elymus nutans* [26] and high AM responsive legume *Trifolium repens* [27,28] were used as host plants. *Elymus nutans* and *T. repens* are good hosts for *P. kansuensis*, and their average shoot dry weight suppression induced by parasitism was 50.16% and 60.44%, respectively [29]. To assess parasitized host responses to AM colonization, we measured a number of host and hemiparasite characteristics, mainly including biomass, photosynthesis, and nutrient status. This study aims to address the following questions: (1) Does *T. repans* benefit more from AM colonization than *E. nutans* in the presence of *P. kansuensis*? (2) Does AM colonization have different effects on the growth of hemiparasitic *P. kansuensis* attached to different host plants? The knowledge obtained will help us better understand the tritrophic interaction of AM fungi, parasitic plants, and host plants and enable us to objectively assess the function of AM fungi on alleviating host damage by hemiparasitic plants.

## 2. Materials and Methods

### 2.1. Experimental Design

*Pedicularis kansuensis* is a widely distributed root hemiparasitic weed in China and is severely spread in most areas of the Bayanbulak Grassland (42°52′48″ N & 83°42′12″ E, 2472 m), Xinjiang Uygur Autonomous Region, China. *Elymus nutans* (grass) and *Trifolium repens* (legume), two common pasture species used for grassland restoration in Bayanbulak Grassland, were selected as host plants in this pot experiment. Four treatments were set up: (1) one hemiparasite with one grass host without AM fungi; (2) one hemiparasite with one grass host with AM fungi; (3) one hemiparasite with one legume host without AM fungi; and (4) one hemiparasite with one legume host with AM fungi. Each treatment had ten replicates.

### 2.2. Plant Materials and Inocula

The seeds of *E. nutans* and *P. kansuensis* were all collected from Bayanbulak Grassland in late August 2010 and late September 2011, respectively, and stored at room temperature in Kunming Institute of Botany, Chinese Academy of Science (KIB), Yunnan Province, China. The seeds of *T. repens* were collected from Kunming Botanical Garden (KBG) of KIB (25°01′ N & 102°41′ E, 1990 m) in 2010 and stored at 4 °C until use. All seeds were soaked in 4.5% sodium hypochlorite for 5–8 min and rinsed thoroughly with distilled water. Germination was carried out on moistened filter papers in *Petri* dishes in an 18/25 °C incubator. The photoperiod was 12 h light with a light intensity of 22.2 µmol photons m^−2^ s^−1^ and 12 h dark.

*Glomus mosseae* (Nicol. And Gerd.) Gerdemann and Trappe (BGC YN05) was used as AM fungus. The inoculum of *G. mosseae*, consisting of colonized root fragments, soil, and spores, was provided by the Institute of Plant Nutrition, Resources and Environment, Beijing Academy of Agriculture and Forestry Sciences. Twenty-one grams of inoculum was added to mycorrhizal pots at ca. 5 cm depth via a tube to reduce seedling disturbance two weeks after transplanting *P. kansuensis* seedlings. The nonmycorrhizal pots received 21 g of the sterilized (121 °C, 30 min) fungal inoculum.

### 2.3. Planting and Growth Conditions

The uniform, newly germinated seeds of hosts and hemiparasites were used. One host seedling was embedded in the pot center. Each pot (1.4 L) contains a 2.1 kg soil mixture of 10% soil collected from KBG and 90% fine sand. The mixture was autoclaved at 121 °C for 2 h before use. The growth medium had a low nutrient content, containing approximately 14.3 mg/kg AN, 2.7 mg/kg AP, and 62.4 mg/kg AK (pH = 6.2). Fourteen days later, five *P. kansuensis* seedlings were transplanted ca. 1.5 cm away from the host. All plants were grown in a simple greenhouse protected with a glass roof and a fly net in KBG. Twenty milliliters of modified Hoagland Nutrient Solution (for the nutrient composition, see Sui et al. [30]) was supplied to each pot weekly after one week of *P. kansuensis* planting. Based on evaporation, all pots were watered by a spry automatic system for half an hour once or twice a day. Autoclaved polyethylene beads were put on the soil surface to reduce moisture loss. Pots in all treatments were randomly moved weekly to reduce position effects.

### 2.4. Harvest and Sampling

The pot experiment was conducted from mid-March to late August (about 24 weeks) in 2012. At harvest, shoots were separated from roots on the soil surface. The shoot and root dry weight (DW), and total DW per plant of *P. kansuensis* and *T. repens* were weighted after oven drying 48 h at 85 °C. AM colonization levels (AMF%) in the roots of host plants and hemiparasites in legume-parasite association were conducted by the magnified intersection method [31] after being treated with 10% KOH and then stained by trypan blue. The AMF%, shoot DW, root DW, and total DW of host and hemiparasite in *E. nutan*-*P. kansuensis* association were taken from Sui et al. [30]. The number of total haustoria (HN) produced by *P. kansuensis* in legume-parasite or grass-parasite associations was counted for each pot. Random parts of haustoria were cleared with 10% KOH and then stained with trypan blue. The number of functional haustoria (with distinct xylem bridges) was counted, and the proportion of functional haustoria (PFH) in HN was calculated. The total number of presumably functional haustoria (FHN) was calculated by the HN times PFH.

### 2.5. Photosynthesis and Chlorophyll Content Analysis

Three pots were randomly chosen to measure the photosynthetic capacity and chlorophyll content (Chla+b) for each treatment. Rapid light response curves under gradient illumination intensity were measured by a MAXI-Imaging Pulse-Amplitude Modulation (PAM) fluorometer (Heinz Walz, Effeltrich, Germany). Before measuring, individuals of *P. kansuensis*, *E. nutans*, and *T. repens* were kept in the dark for more than 20 min. The relative electron transfer rate (ETR) was obtained from fresh and detached leaves using a chlorophyll fluorometer [32]. The chlorophylls of hosts and hemiparasites leaves were extracted by N, N-dimethyl formamide (for details, see Sui et al. [33]). Total chlorophyll contents were calculated as described by Minocha et al. [34] and Porra et al. [35] based on the light absorption values of chlorophyll under 664 nm and 647 nm.

### 2.6. Shoot N and P Analysis

For each treatment, three replicate pots were also randomly chosen to do shoot N and P analysis. Dried shoot tissues of *P. kansuensis*, *E. nutans*, and *T. repens* were separately ground and digested with a sulfuric–salicylic acid mix [36]. Shoot N concentrations were determined by distillation in a Kjeldahl apparatus (BUCHI K360, Flawil, Switzerland). Shoot P concentrations were obtained by the method of phosphovanado-molybdate [37] and a spectrophotometer (UV1601 Shimadzu, Kyoto, Japan). The element concentration times shoot DW is the total shoot N or P content (mg per plant).

### 2.7. Data Analysis

Only data from pots with both host plants and the hemiparasite that survived at harvest in all treatments were used to perform subsequent biomass and haustoria analyses. Three replicates were used to perform the analysis of AMF%, nutrient content, and chlorophyll content. Two independent sample *t* tests were used to analyze the effect of AM colonization on shoot DW, root DW, total DW, shoot N and P concentrations (contents), total chlorophyll content (Chla+b), ETR of *P. kansuensis* and host plants, HN and FHN formed by *P. kansuensis*. Two-way ANOVAs (*p* ≤ 0.05) were used to analyze the effect of host species and AM colonization on shoot DW, root DW, total DW, HN, FHN, shoot N and P content, and total chlorophyll content of *P. kansuensis*. The effect size was indicated by partial eta squared (η_p_^2^). All data were enforced the normal distribution test or homogeneity test of variances before analyses. Data for shoot DW, total DW, and leaf Chla+b of *Pedicularis* were log_10_ transformed to conform normality and homogeneity of residuals. All analyses were conducted in Statistical Product and Service Solution (SPSS, version 16.0, SPSS China Ltd., Shanghai, China).

## 3. Results

### 3.1. AM Colonization and Haustorium Formation

The AM colonization level of parasitized *T. repens* was higher than that of parasitized *E. nutans* (Table 1). The AM colonization level of *P. kansuensis* was lower in the legume-parasite association than in the grass-parasite association (Table 1). No AM colonization was observed in non-inoculated roots of grass, legume, and hemiparasite.

Host species and AM inoculation showed a significant two-way interactive effect on the number of functional haustoria (FHN) produced by *P. kansuensis* (*p* = 0.005, η_p_^2^ = 0.402; Table 2), but they had no interactive effect on the total number of haustoria (HN) (*p* = 0.057, η_p_^2^ = 0.208; Table 2). Although AM inoculation had no independent effect on FHN (*p* = 0.054, η_p_^2^ = 0.212; Table 2), it significantly decreased FHN in *P. kansuensis* in grass-hemiparasite pairs (t = 3.07, *p* = 0.01, Table 1). FHN produced by *P. kansuensis* was increased (but not significantly) by AM colonization (73.71%, t = −1.68, *p* = 0.18, Table 1) in legume-parasite pairs.

### 3.2. Growth Performance of Host Plants and Hemiparasite

**Figure 1:**
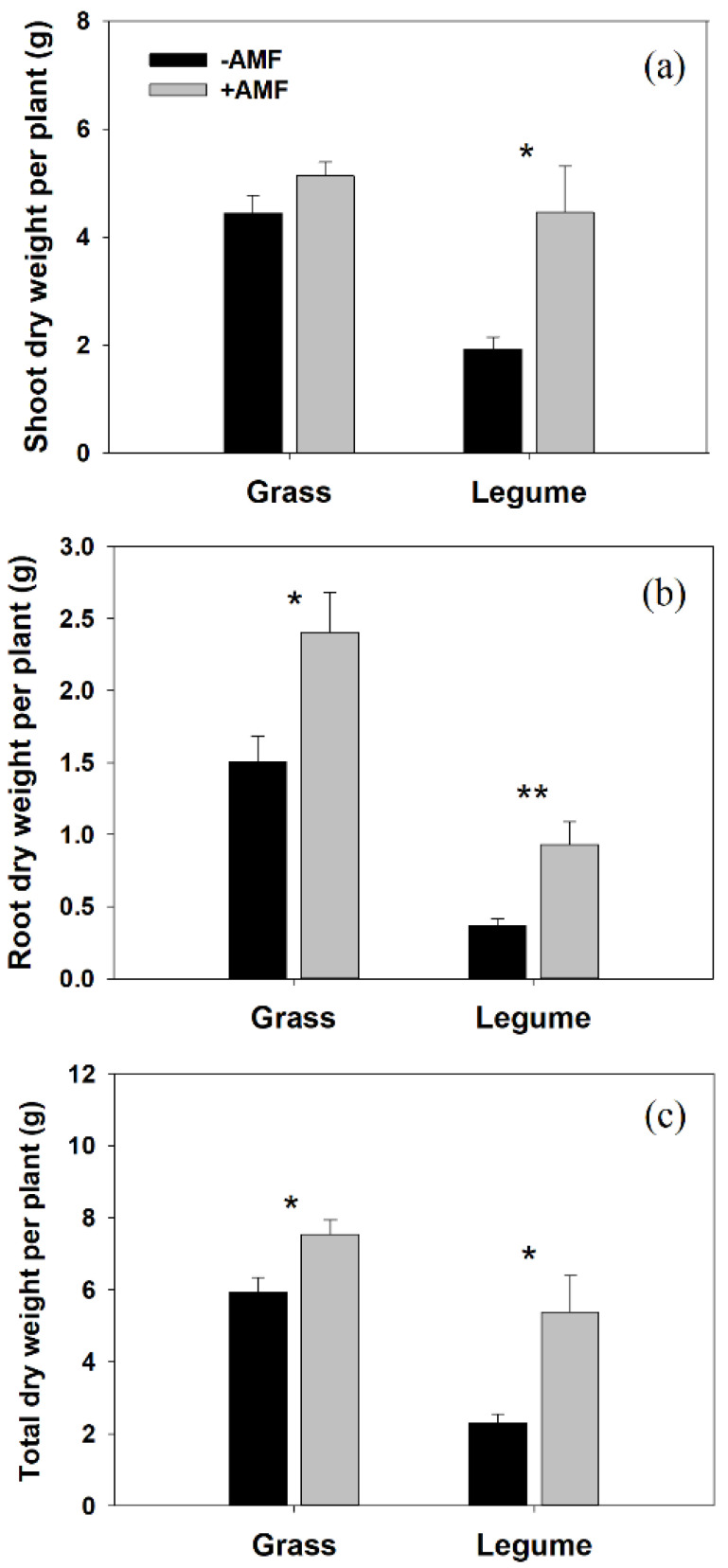
Shoot dry weight (DW), root DW, and total DW of host plants (**a**–**c**). Data are presented as the mean ± standard error. There are seven replicates for the nonmycorrizal grass-hemiparasite association, five replicates for the nonmycorrizal legume-hemiparasite association, and four replicates for the mycorrizal grass-hemiparasite and legume-hemiparasite associations. Grass: *Elymus nutans*; Legume: *Trifolium repens*. Significant differences (as determined by *t* test) between mycorrhizal (gray bars) and non-mycorrhizal (black bars) treatments are indicated by asterisks (* *p* < 0.05, ** *p* < 0.01). Biomass data of *E. nutans* were taken from Sui et al. [30].

Inoculation with AM fungi significantly improved shoot DW (131.65%; t = 3.165, *p* = 0.016), root DW (151.27%; t = 3.803, *p* = 0.007), and total DW (134.82%; t = 3.354, *p* = 0.012) of *T. repens* parasitized by *P. kansuensis*. AM inoculation increased shoot DW (15.73%; t = 1.641, *p* = 0.135), root DW (60.15%; t = 2.843, *p* = 0.019), and total DW (26.96%; t = 2.623, *p* = 0.028) of parasitized *E. nutans* (Figure 1a–c).

**Figure 2:**
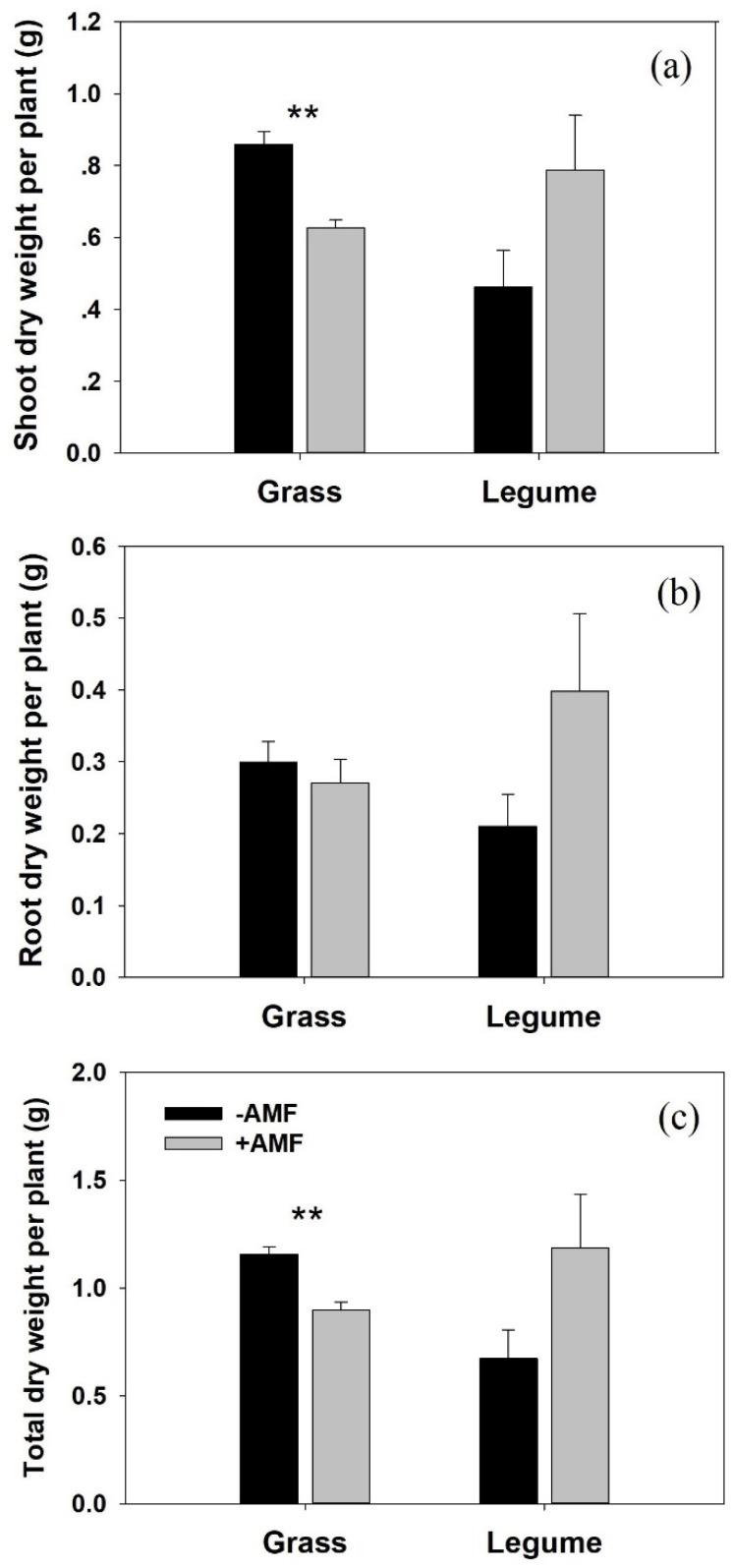
Shoot dry weight (DW), root DW, and total DW of *Pedicularis kansuensis* (**a**–**c**). Data are presented as the mean ± standard error. There are seven replicates for the nonmycorrhizal grass-hemiparasite association, five replicates for the nonmycorrizal legume-hemiparasite association, and four replicates for the mycorrizal grass-hemiparasite and legume-hemiparasite associations. Grass: *Elymus nutans*; Legume: *Trifolium repens*. Significant differences (as determined by *t*-test) between mycorrhizal (gray bars) and non-mycorrhizal (black bars) treatments are indicated by asterisks (** *p* < 0.01). Biomass data of *P. kansuensis* were taken from Sui et al. [30].

Host species and AM inoculation showed significant interaction effects on the shoot DW (*p* = 0.005, η_p_^2^ = 0.397; Table 2) and total DW (*p* = 0.016, η_p_^2^ = 0.311; Table 2) of *P. kansuensis*. Inoculation with AM fungi significantly suppressed the shoot DW (t = 4.431, *p* = 0.002) and total DW (t = 5.101, *p* = 0.001) of *P. kansuensis* when grown with *E. nutans*. The shoot DW, root DW, and total DW of *P. kansuensis* grown with mycorrhizal white clover were all higher than those grown with nonmycorrhizal white clover (Figure 2a–c), but there was no significance (Figure 2). Host species had no significant independent effect on the growth performance of *P. kansuensis* (Table 2).

### 3.3. Tissue Element Concentration of Hosts and Hemiparasite

**Figure 3:**
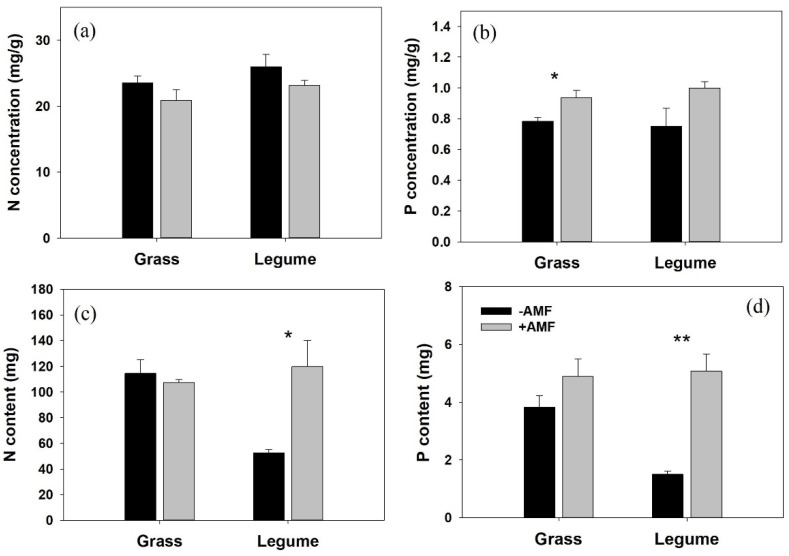
Nitrogen (N, (**a**)) and phosphorus (P, (**b**)) concentrations (mg/g) and contents (mg per plant, N, (**c**) and P, (**d**)) in shoots of *Elymus nutans* (Grass) and *Trifolium repens* (Legume). Data are presented as the mean ± standard error of three replicated pots. Significant differences (as determined by *t* test) between mycorrhizal (gray bars) and nonmycorrhizal (black bars) treatments are indicated by asterisks (* *p* < 0.05, ** *p* < 0.01).

The shoot P concentration of grass hosts in the mycorrhizal pots was much higher than that in nonmycorrhizal pots (Figure 3b). However, the shoot N concentration, shoot N and P contents of mycorrhizal *E. nutans* were not apparently different from those of nonmycorrhizal *E. nutans* (Figure 3a,c,d). Inoculation with AM fungi had little effect on shoot N and P concentrations of legume hosts. Nevertheless, it significantly improved the contents of shoot N and P in parasitized legume hosts (Figure 3c,d).

**Figure 4:**
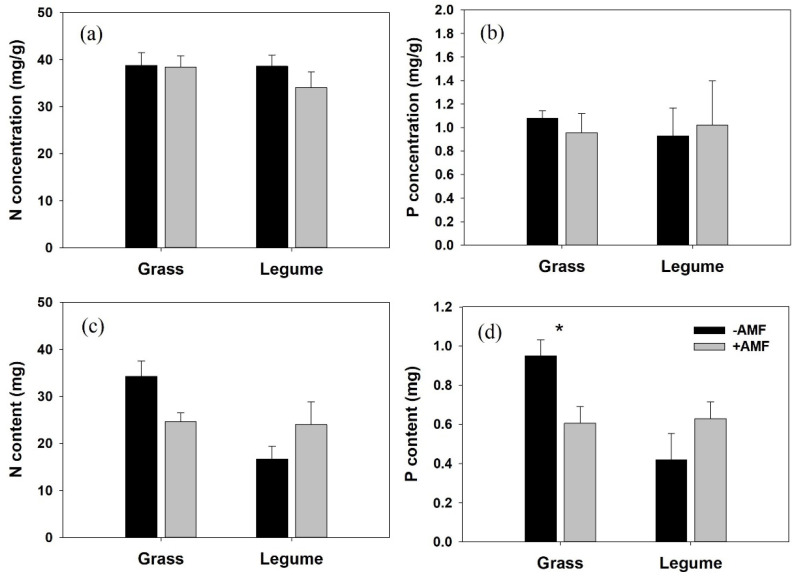
Nitrogen (N, (**a**)) and phosphorus (P, (**b**)) concentrations (mg/g) and contents (mg per plant, N, (**c**) and P, (**d**)) in shoots of *Pedicularis kansuensis*. Data are presented as the mean ± standard error of three replicated pots. Significant differences (as determined by *t* test) between mycorrhizal (gray bars) and nonmycorrhizal (black bars) treatments are indicated by asterisks (* *p* < 0.05).

The host species and AM inoculation showed significant interactive effects on shoot N (*p* = 0.036, η_p_^2^ = 0.443) and P (*p* = 0.023, η_p_^2^ = 0.494) contents of *P. kansuensis* (Table 3). The host species independently and significantly affected shoot N (*p* = 0.027, η_p_^2^ = 0.478; Table 3) and P (*p* = 0.034, η_p_^2^ = 0.449) contents of *P. kansuensis*, but AM inoculation had no significant effect on the shoot N and P contents of *P. kansuensis* (Table 3). In the nonmycorrhizal treatment, *Pedicularis* shoot N and P contents were substantially higher when attached to grass hosts than when attached to legume hosts (Figure 4c,d). Inoculation with AM fungi significantly reduced the shoot P content of *P. kansuensis* grown with grass hosts (t = 2.906, *p* = 0.04), but slightly improved shoot N and P contents of *P. kansuensis* parasitizing legumes (Figure 4c,d). Both host species and AM fungi had no statistically significant effect on the shoot N and P concentrations of *P. kansuensis* (Figure 4a,b, Table 3).

### 3.4. Photosynthetic Capacity of Host Plants and P. kansuensis

**Figure 5:**
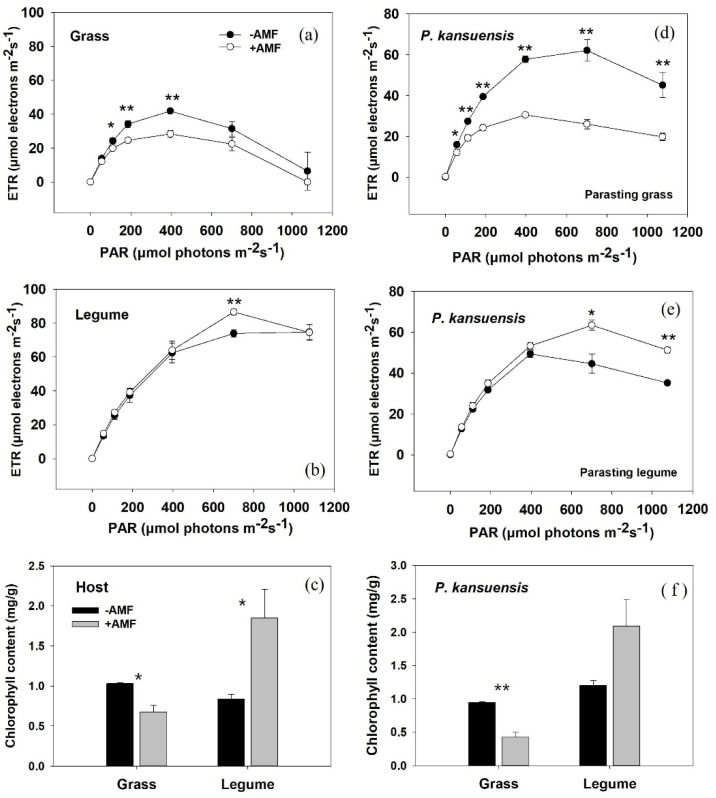
Electron transport rate (ETR) light-response curves and total chlorophyll contents in leaves of *Elymus nutans* (grass, (**a**,**c**)), *Trifolium repens* (legume, (**b**,**c**)) and *Pedicularis kansuensis* (**d**–**f**). Data are presented as the mean ± standard error of three replicated pots. Significant differences (as determined by *t* test) between mycorrhizal (gray bars) and nonmycorrhizal (black bars) treatments are indicated by asterisks (* *p* < 0.05, ** *p* < 0.01).

For hosts parasitized by *P. kansuensis*, *E. nutans* exhibited significantly lower ETR values in mycorrhizal pots than in nonmycorrhizal pots (Figure 5a). The ETR values of parasitized *T. repens* were higher in mycorrhizal pots than in nonmycorrhizal pots only at 721 μmol m^−2^ s^−1^ photosynthetically active radiation (Figure 5b). Inoculation with AM fungi significantly suppressed the ETR of *P. kansuensis* in the parasite-grass association (Figure 5d), but greatly improved the ETR of *P. kansuensis* in the parasite-legume association (Figure 5e).

Host species and AM inoculation had a significant interactive impact on the total chlorophyll contents of *P. kansuensis* (*p* = 0.001 η_p_^2^ = 0.770; Table 3). The total chlorophyll contents of both *T. repens* and *P. kansuensis* were significantly increased by AM inoculation, especially for the parasitized *T. repens* (Figure 5c,f). In contrast, inoculation with AM fungi greatly reduced the total chlorophyll contents of both host and parasite in the *E. nutans-P. kansuensis* association (Figure 5c,f).

## 4. Discussion

**Figure 6:**
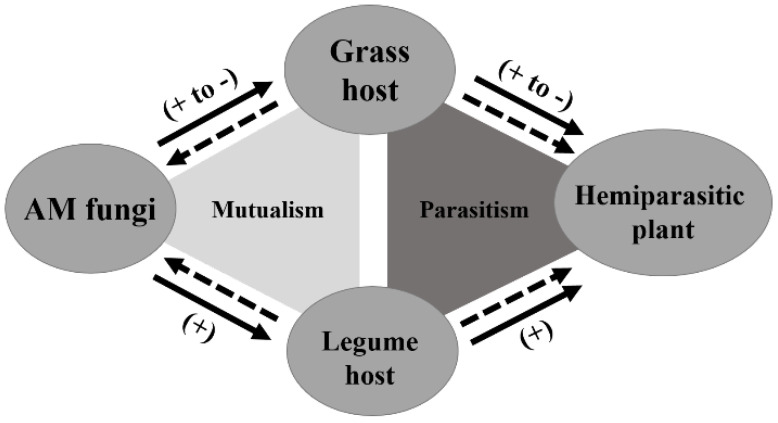
Simple model of interaction among AM fungi, grass host, legume host, and hemiparasitic plant. The arrow direction indicates the direction of nutrient flow. Solid arrows represent mineral nutrient flow, and dashed arrows indicate carbon flow. The plus and minus in parentheses represent directly positive or negative effects of AM fungi on the growth of different host plants and the indirect effect of AM fungi on the growth of hemiparasites attached to grass or legume hosts.

Mutualism and parasitism are two typical ecological interactions, but have different effects on interspecific relationships. In general, parasitic plants are detrimental to plant growth, while AM fungi are beneficial. The struggle between the beneficial AM fungi and the harmful hemiparasitic plants codetermines the growth performance of host plants. A simplified model of AM fungi’s direct and indirect effects on the growth of different host plants and hemiparasitic plants is presented as a framework (Figure 6).

Our results confirmed that the host’s responses to AM colonization determined the effect of AM fungi on the performance of parasitized host plants and proved that higher AM responsive legume benefits more from AM colonization than less AM responsive grass in the presence of hemiparasitic plants. The growth performance of the parasitic plant is positively correlated with host damage, as a sizeable hemiparasitic plant can cause severe damage to the host plant [38,39,40]. In this study, we found that although the total DW of the *P. kansuensis* parasitizing legume host was higher in the AM treatment than the NM treatment, the growth performance of *T. repens* (shoot DW improvement, 131.65%) was still greatly improved by AM colonization. In contrast, despite the shoot DW of the *P. kansuensis* parasitizing grass host decreased significantly in the AM treatment, the shoot DW improvement of *E. nutans* was only 15.73%. These results indicated that the positive effect of AM fungi on the growth of *T. repens* is enough to compensate for the negative impact of parasitism by *P. kansuensis*. However, this is not the case in the grass-hemiparasite association.

Grasses and legumes have a low ability to resist the invasion of haustoria and have been proven to be good hosts for most hemiparasitic plants [38,41], but generally have different responses to AM colonization. Although many abiotic or biotic factors have been reported to influence the effect of AM fungi on plant growth [23], previous studies have shown that root morphology modifies the responses of plants to AM colonization [21]. For example, taproot plants (such as legumes) showed a more fantastic growth response to colonization by AM fungi than plants with fibrous root systems (such as grass, especially C_3_ grass) [21,42]. In this study, we did not test the effect of AM fungi on host growth in the absence of a parasite. However, the different effects of *G. mosseae* on the growth of *E. nutans* and *T. repens* have been demonstrated previously. Numerous studies showed the significant positive impact of *G. mosseae* on the growth of *T. repens* via enhancing its nutrient status [27,28,43]. In contrast, inoculation with *G. mosseae* had little effect on the shoot DW of *E. nutans* [26,44,45,46], but the outcomes differed in cultivars [26] and were affected by soil nitrogen forms [46] or temperature [45]. In this experiment, we found that the excellent growth improvement of parasitized *T. repens* by AM fungi was attributed not only to the increase in root absorption capacity (shoot N and P content, Figure 3c,d) but also to the enhancement of whole-plant photosynthetic capacity (unit rate × leaf area) (Figure 1a and Figure 5b). In contrast, inoculation with AM fungi significantly suppressed the ETR of parasitized *E. nutans* (Figure 5a), but slightly improved the shoot DW of the parasitized grass host (Figure 1c). This phenomenon can be explained by the inhibited growth of *P. kansuensis* parasitizing mycorrhizal *E. nutans*.

The growth responses of *P. kansuensis* to AM colonization varied in different host treatments. In this study, we found that AM colonization significantly improved the growth performance of the parasitic plant in the legume-parasite association (Figure 2). This result is consistent with that obtained by Salonen et al. [14], who found that the growth performance of *R. serotinus* was improved when associated with mycorrhizal *T. pretense* (Legume). In that study, the authors realized that hemiparasite growth performance was affected by the AM status of host plants, but they could not clearly explain the mechanism. In the present study, we found that the growth stimulation of *P. kansuensis* grown with *T. repens* in the presence of AM fungi was most likely related to two processes. First, connected to mycorrhizal *T. repens*, *P. kansuensis* obtained access to a larger and robust host (Figure 1), gathering more mineral nutrients from the soil and producing more carbohydrates. More haustoria produced by *P. kansuensis* in mycorrhizal pots was correlated with more nutrient availability from host to parasite and then stimulated the growth of *P. kansuensis*. Second, the increased photosynthetic capacity (higher ETR and Chla+b, Figure 5e,f) of *P. kansuensis* grown with mycorrhizal *T. repens* partly facilitated parasite growth. In contrast, inoculation with *G. mosseae* significantly reduced the shoot DW of *P. kansuensis* attached to *E. nutans* (Figure 2a). This can be partially explained by the inhibited photosynthetic capacity (lower ETR and total chlorophyll content, Figure 5d,f) of the hemiparasitic plant when attached to mycorrhizal *E. nutans*. The reduction in photosynthetic capacity indicated a decrease in photosynthate (C) production, hence inhibiting the formation of energy-costed haustoria [7,47]. As expected, a fewer number of haustoria (FHN decrement, 89.36%) was correlated with the lower shoot N and P content of *P. kansuensis* attached to mycorrhizal *E. nutans* (Figure 4c,d). These results indicated that AM colonization suppressed the formation of haustoria, and hence, the nutrient transport from the host to the parasite in this grass-parasite association. The negative effect of AM fungi on hemiparasitic plants grown with grass hosts has been shown in other grass-hemiparasite associations. For example, Li et al. [48] found that inoculation with AM fungi significantly suppressed the haustoria formation and growth performance of *Pedicularis rex* or *P. tricolor* when attached to *Hordeum vulgare*. However, Davies and Graves [7] found inoculation with AM fungi improved the growth and haustorium formation of *R. minor* attached to mycorrhizal *Lolium perenne*. The different effects of AM colonization on the growth and haustoria formation of *Pedicualris* and *Rhinanthus* in grass-parasite pairs may be due to their different responses to AM colonization. The former can directly connect with AM fungi [49], but the latter was confirmed as a nonmycorrhizal plant [25,50]. However, further investigations are required.

In this study, we proved that the higher AM responsive host plant benefits more from AM colonization in the presence of hemiparasitic plants and set out the mechanisms. However, there are still many unclear questions regarding the effect of AM fungi on the interaction of root hemiparasites and their host plants. For example, in this study, we found different influences of AM colonization on the growth performance of hemiparasitic plants grown with grass or legume hosts. The cocultivation system (AM fungi, host, and parasite coexisting in one pot) made it difficult to distinguish the effect of AM fungi on the growth of parasites directly or indirectly (via host plant). In the future, a split-root system and multi-compartment box experiment can be conducted to investigate this puzzle.

## 5. Conclusions

The effect of AM fungi on the interactions of hemiparasite-grass hosts and hemiparasite-legume hosts was comparatively investigated for the first time. Our results demonstrated the essential role of AM fungi in alleviating host damage induced by hemiparasitic plants and found that higher AM responsive host benefits more from AM colonization. The results also confirmed the earlier hypothesis that the AM status of host plants could affect the performance of attached root hemiparasites. Grasses and legumes are common species in the plant community and preferred host species for most hemiparasites. The different influences of AM fungi on the interactions of legume-hemiparasite and grass-hemiparasite indicated that changes in interspecies competition among grasses, legumes, and parasites (and forbs) should be found in the mycorrhizal environment. Further work needs to take AM fungi into account when addressing the effect of hemiparasitic plants on community structure and community productivity.

## Figures and Tables

**Table 1:** AM colonization level (AMF%) of host and *Pedicularis kansuensis* and number of huastoria (HN) and number of functional haustoria (FHN) produced by *P. kansuensis* in different treatments.

Host Species	AMF Treatment	AMF%		HN	FHN
		Host	*P. kansuensis*		
**Grass**	−AMF	0	0	387.29 ± 88.90 a	252.07 ± 53.51 a
	+AMF	82.60 ± 1.40% *	49.50 ± 3.20% *	89.50 ± 50.53 b	26.80 ± 15.97 b
**Legume**	−AMF	0	0	125.00 ± 37.15	68.53 ± 6.43
	+AMF	96.67 ± 2.74%	27.69 ± 4.08%	125.50 ± 32.75	119.04 ± 29.34

Data are mean ± standard error of three replicates for AMF%, seven replicates for HN and FHN in the nonmycorrizal grass-hemiparasite association, five replicates for HN and FHN in the nonmycorrizal legume-hemiparasite association, and four replicates for HN and FHN in the mycorrhizal grass-hemiparasite and legume-hemiparasite associations. Different letters denote significant differences at *p* < 0.05 between the two AM fungal treatments based on two independent samples *t* test. * means the data were taken from Sui et al. [30].

**Table 2:** Two-way ANOVA results (η_p_^2^ values and *p* values) for the effects of host species (Hsp) and AM colonization (AM) on shoot DW and root DW, total DW, number of haustoria (HN), and functional haustoria (FHN) of *Pedicularis kansuensis*.

		Shoot DW		Root DW		Total DW		HN		FHN	
Effect	df	η_p_^2^	*p*	η_p_^2^	*p*	η_p_^2^	*p*	η_p_^2^	*p*	η_p_^2^	*p*
**Hsp**	1,20	0.116	0.167	0.008	0.731	0.113	0.172	0.131	0.140	0.069	0.294
**AM**	1,20	0.021	0.564	0.118	0.164	0.060	0.327	0.206	0.058	0.212	0.054
**Hsp × AM**	1,20	**0.397**	**0.005**	0.198	0.064	**0.311**	**0.016**	0.208	0.057	**0.402**	**0.005**

Values suggesting significant effects (*p* < 0.05) are given in bold.

**Table 3:** Two-way ANOVA results (η_p_^2^ values and *p* values) for the effects of host species (Hsp) and AM colonization (AM) on shoot N concentration, P concentration, N content, P content and chlorophyll content (Chla+b) of *Pedicularis kansuensis*.

		N Concentration		P Concentration		N Content		P Content		Chla+b	
Effect	df	η_p_^2^	*p*	η_p_^2^	*p*	η_p_^2^	*p*	η_p_^2^	*p*	η_p_^2^	*p*
**Hsp**	1,12	0.080	0.427	0.004	0.865	**0.478**	**0.027**	**0.449**	**0.034**	**0.862**	**0.000**
**AM**	1,12	0.093	0.392	0.001	0.950	0.014	0.741	0.054	0.519	0.139	0.288
**Hsp** **×** **AM**	1,12	0.070	0.461	0.024	0.670	**0.443**	**0.036**	**0.494**	**0.023**	**0.770**	**0.001**

Values suggesting significant effects (*p* < 0.05) are given in bold.

## Data Availability

Not applicable.

## References

[1] Bardgett R.D., Smith R.S., Shiel R.S., Peacock S., Simkin J.M., Quirk H., Hobbs P.J. (2006). Parasitic plants indirectly regulate below-ground properties in grassland ecosystems. Nature.

[2] Demey A., De Frenne P., Baeten L., Verstraeten G., Hermy M., Boeckx P., Verheyen K. (2015). The effects of hemiparasitic plant removal on community structure and seedling establishment in semi-natural grasslands. J. Veg. Sci..

[3] Press M.C., Phoenix G.K. (2005). Impacts of parasitic plants on natural communities. New Phytol..

[4] Quested H.M. (2008). Parasitic plants-impacts on nutrient cycling. Plant Soil.

[5] Jiang F., Jeschke W.D., Hartung W., Cameron D.D. (2010). Interactions between *Rhinanthus minor* and its hosts: A review of water, mineral nutrient and hormone flows and exchanges in the hemiparasitic association. Folia Geobot..

[6] Joel D.M., Gressel J., Musselman L.J. (2013). Parasitic Orobanchaceae-Parasitic Mechanisms and Control Strategies.

[7] Davies D.M., Graves J.D. (1998). Interactions between arbuscular mycorrhizal fungi and the hemiparasitic angiosperm *Rhinanthus minor* during co-infection of a host. New Phytol..

[8] Irving L.J., Cameron D.D. (2009). You are what you eat: Interactions between root parasitic plants and their hosts. Advances in Botanical Research.

[9] Tesitel J., Leps J., Vrablova M., Cameron D.D. (2011). The role of heterotrophic carbon acquisition by the hemiparasitic plant *Rhinanthus alectorolophus* in seedling establishment in natural communities: A physiological perspective. New Phytol..

[10] Cirocco R.M., Facelli J.M., Watling J.R. (2017). Does nitrogen affect the interaction between a native hemiparasite and its native or introduced leguminous hosts?. New Phytol..

[11] Cirocco R.M., Facelli J.M., Watling J.R. (2016). High water availability increases the negative impact of a native hemiparasite on its non-native host. J. Exp. Bot..

[12] Cirocco R.M., Facelli E., Delean S., Facelli J.M. (2021). Does phosphorus influence performance of a native hemiparasite and its impact on a native legume?. Physiol. Plant..

[13] Cechin I., Press M.C. (1994). Influence of nitrogen on growth and photosynthesis of a C_3_ cereal, *Oryza sativa*, infected with the root hemiparasite *Striga hermonthica*. J. Exp. Bot..

[14] Salonen V., Vestberg M., Vauhkonen M. (2001). The effect of host mycorrhizal status on host plant-parasitic plant interactions. Mycorrhiza.

[15] Sui X.L., Zhang T., Tian Y.T., Xue R.J., Li A.R. (2019). A neglected alliance in battles against parasitic plants: Arbuscular mycorrhizal and rhizobial symbioses alleviate damage to a legume host by root hemiparasitic *Pedicularis* species. New Phytol..

[16] Kapoor R., Sharma D., Bhatnagar A.K. (2008). Arbuscular mycorrhizae in micropropagation systems and their potential applications. Sci. Hortic..

[17] Smith S.E., Smith F.A. (2011). Roles of arbuscular mycorrhizas in plant nutrition and growth: New paradigms from cellular to ecosystem scales. Annu. Rev. Plant Biol..

[18] Chen X., Tang J.J., Zhi G.Y., Hu S.J. (2005). Arbuscular mycorrhizal colonization and phosphorus acquisition of plants: Effects of coexisting plant species. Appl. Soil Ecol..

[19] Sabais A.C.W., Eisenhauer N., Konig S., Renker C., Buscot F., Scheu S. (2012). Soil organisms shape the competition between grassland plant species. Oecologia.

[20] Liu B., Li H., Zhu B., Koide R.T., Eissenstat D.M., Guo D. (2015). Complementarity in nutrient foraging strategies of absorptive fine roots and arbuscular mycorrhizal fungi across 14 coexisting subtropical tree species. New Phytol..

[21] Yang H.S., Zhang Q., Dai Y.J., Liu Q., Tang J.J., Bian X.M., Chen X. (2015). Effects of arbuscular mycorrhizal fungi on plant growth depend on root system: A meta-analysis. Plant Soil.

[22] Irving L.J., Kim D., Schwier N., Vaughan J.K.E., Ong G., Hama T. (2019). Host nutrient supply affects the interaction between the hemiparasite *Phtheirospermum japonicum* and its host *Medicago sativa*. Environ. Exp. Bot..

[23] Jin L., Wang Q., Wang Q., Wang X.J., Gange A.C. (2017). Mycorrhizal-induced growth depression in plants. Symbiosis.

[24] Niemelä M., Markkola A., Mutikainen P. (2008). Modification of competition between two grass species by a hemiparasitic plant and simulated grazing. Basic Appl. Ecol..

[25] Stein C., Rissmann C., Hempel S., Renker C., Buscot F., Prati D., Auge H. (2009). Interactive effects of mycorrhizae and a root hemiparasite on plant community productivity and diversity. Oecologia.

[26] Chu X.T., Fu J.J., Sun Y.F., Xu Y.M., Miao Y.J., Xu Y.F., Hu T.M. (2016). Effect of arbuscular mycorrhizal fungi inoculation on cold stress-induced oxidative damage in leaves of *Elymus nutans* Griseb. S. Afr. J. Bot..

[27] Cao S., Ren A., Lu W. (2015). Effect of inoculation with AM fungi on four forages growth. Grassl. Turf.

[28] Jongen M., Fay P., Jones M.B. (1996). Effects of elevated carbon dioxide and arbuscular mycorrhizal infection on *Trifolium repens*. New Phytol..

[29] Sui X.L. (2014). Exo-Physiological Mechanism for the Spatial Expansion of *Pedicularis kansuensis* in Bayanbulak Grassland of Xinjiang. Ph.D. Thesis.

[30] Sui X.L., Li A.R., Chen Y., Guan K.Y., Zhuo L., Liu Y.Y. (2014). Arbuscular mycorrhizal fungi: Potential biocontrol agents against the damaging root hemiparasite *Pedicularis kansuensis*?. Mycorrhiza.

[31] Mcgonigle T.P., Miller M.H., Evans D.G., Fairchild G.L., Swan J.A. (1990). A new method which gives an objective-measure of colonization of roots by vesicular arbuscular mycorrhizal fungi. New Phytol..

[32] White A.J., Critchley C. (1999). Rapid light curves: A new fluorescence method to assess the state of the photosynthetic apparatus. Photosynth. Res..

[33] Sui X.L., Huang W., Li Y.J., Guan K.Y., Li A.R. (2015). Host shoot clipping depresses the growth of weedy hemiparasitic *Pedicularis kansuensis*. J. Plant Res..

[34] Minocha R., Martinez G., Lyons B., Long S. (2009). Development of a standardized methodology for quantifying total chlorophyll and carotenoids from foliage of hardwood and conifer tree species. Can. J. For. Res..

[35] Porra R.J., Thompson W.A., Kriedemann P.E. (1989). Determination of accurate extinction coefficients and simultaneous-equations for assaying chlorophyll-a and chlorophyll-b extracted with 4 different solvents -verification of the concentration of chlorophyll standards by atomic-absorption spectroscopy. Biochim. Biophys. Acta.

[36] Allen S.E., Grimshaw H.M., Parkinson J.A., Quarmby C. (1974). Chemical Analysis of Ecological Materials.

[37] Hanson W.C. (1950). The photometric determination of phosphorus in fertilizers using the phosphovanado-molybdate complex. J. Sci. Food Agric..

[38] Cameron D.D., Seel W.E. (2007). Functional anatomy of haustoria formed by *Rhinanthus minor*: Linking evidence from histology and isotope tracing. New Phytol..

[39] Hwangbo J.K., Seel W.E., Woodin S.J. (2003). Short-term exposure to elevated atmospheric CO_2_ benefits the growth of a facultative annual root hemiparasite, *Rhinanthus minor* (L.), more than that of its host, *Poa pratensis* (L.). J. Exp. Bot..

[40] Matthies D. (2017). Interactions between a root hemiparasite and 27 different hosts: Growth, biomass allocation and plant architecture. Perspect. Plant Ecol. Evol. Syst..

[41] Rümer S., Cameron D.D., Wacker R., Hartung W., Jiang F. (2007). An anatomical study of the haustoria of *Rhinanthus minor* attached to roots of different hosts. Flora.

[42] Lin G., McCormack M.L., Guo D. (2015). Arbuscular mycorrhizal fungal effects on plant competition and community structure. J. Ecol..

[43] Wang D., Yang S.M., Tang F., Zhu H.Y. (2012). Symbiosis specificity in the legume-rhizobial mutualism. Cell Microbiol..

[44] Sun Y., Fu J., Chu X., Yang Y., Xu Y., Hu T. (2015). Effects of arbuscular mycorrhizal fungi on the uptake of different nitrogen sources in *Elymus nutans*. Acta Agrestia Sin..

[45] Xu Y., Chu X., Wu Y., Miao Y., Xu Y., Hu T., Xie G. (2016). Effect of arbuscular mycorrhiza inoculation on cold resistance in *Elymus nutans*. Acta Agrestia Sin..

[46] Xu Y.F., Chu X.T., Zhang X.H., Liu Q., Miao Y.J., Sun Y.F. (2018). The forms of nitrogen source influence the interaction between *Elymus nutans* Griseb. and arbuscular mycorrhizal fungi. S. Afr. J. Bot..

[47] Stewart G.R., Press M.C. (1990). The physiology and biochemistry of parasitic angiosperms. Annu. Rev. Plant Phys..

[48] Li A.R., Guan K.Y., Stonor R., Smith S.E., Smith F.A. (2013). Direct and indirect influences of arbuscular mycorrhizal fungi on phosphorus uptake by two root hemiparasitic *Pedicularis* species: Do the fungal partners matter at low colonization levels?. Ann. Bot..

[49] Li A.R., Guan K.Y. (2007). Mycorrhizal and dark septate endophytic fungi of *Pedicularis* species from northwest of Yunnan Province, China. Mycorrhiza.

[50] Lesica P., Antibus R.K. (1986). Mycorrhizal status of hemiparasitic vascular plants in Montana, USA. Trans. Br. Mycol. Soc..

